# Secondary Bilateral Angle Closure Glaucoma due to Topiramate

**DOI:** 10.1155/2011/594051

**Published:** 2011-12-22

**Authors:** Miguel Paciuc-Beja, Myriam Retchkiman-Bret, Cecilio Francisco Velasco-Barona, Victor Hugo Galicia-Alfaro

**Affiliations:** ^1^Eye Clinic, Denver Health and Hospital Authority, Davis Pavillion, Colorado, 777 Bannock Street, Denver, CO 80204, USA; ^2^Department of Ophthalmology, ABC Medical Center, 154 Carlos Graff Fernández, Cuajimalpa, Santa Fe, 05300 Mexico City, Mexico

## Abstract

We examined a 39-year-old female with severe headache and blurred vision. She was on topiramate, 50 mg once a day for one week because of migraine. Periorbital edema, chemosis, myopia, high intraocular pressures, and shallow anterior chambers were present at the initial examination. Iridocorneal angles were closed, ultrasound showed choroidal effusions. We stopped topiramate and started antiglaucoma treatment. After one week the intraocular pressure was 10 mm Hg in both eyes without treatment. A new ultrasound showed no choroidal effusions. Topiramate has been associated with acute secondary angle closure glaucoma as an idiosyncratic reaction to the drug. Physicians prescribing topiramate need to alert patients of this potential sight-threatening idiosyncratic reaction.

## 1. Introduction

Topiramate, a sulfamate-substituted monosaccharide, is primarily used in the treatment of epilepsy, however, it has also demonstrated efficacy in the treatment of migraine, depression, bipolar disorders, neuropathic pain, posttraumatic stress disorder, postherpetic neuropathy, idiopathic intracranial hypertension, and as “off-label application” it has been used for weight loss, adjunctive therapy for alcoholism and nicotine cessation [1–3]. Topiramate is prescribed for adults and pediatric patients aged 2 to 16 years with partial-onset seizures or primary generalized tonic-clonic seizures [4]. 

It was launched into the market in 1996, and only 5 years later Banta et al. [5] reported the first case of acute angle closure glaucoma (AACG). On September 2001, Ortho-McNeil Pharmaceuticals inserted an alert in the box indicating that 23 cases of AACG had been reported to their safety division, and physician should be aware of this adverse ocular drug reaction [6].

The ocular adverse reaction consists of peripheral ciliochoroidal effusion with ciliary body edema and anterior rotation of the ciliary body. There is an anterior shifting of the lens iris diaphragm shallowing the anterior chamber in the periphery causing the AACG without pupillary block. The exact mechanism of the ciliochoroidal effusion is unknown. It is believed to be an idiosyncratic adverse reaction to the drug.

Several drugs have been responsible for sporadic cases of bilateral acute angle closure glaucoma with choroidal effusions secondary to an idiosyncratic reaction [7].

## 2. Case Report

A 39-year-old woman was seen because of headache, blurred vision, and lid edema for the past 2 days. Her prior refraction was OD: +1.75 sph and OS: +1.25 sph, her BCV was 20/20.

At the time of examination, the uncorrected VA was of 20/70 in each eye. A new refraction showed OD: −1.50 = −0.75 × 100, and OS: −2.00 = −1.00 × 110. Her IOP was OD 36 mm Hg, and OS 40 mm Hg, anterior chambers were shallow, pupils were 5 mm and slightly reactive to light, and the optic cup/disc ratio was 0.3 in both eyes. The iridocorneal angle was closed 360 degrees in both eyes.

**Figure 1:**
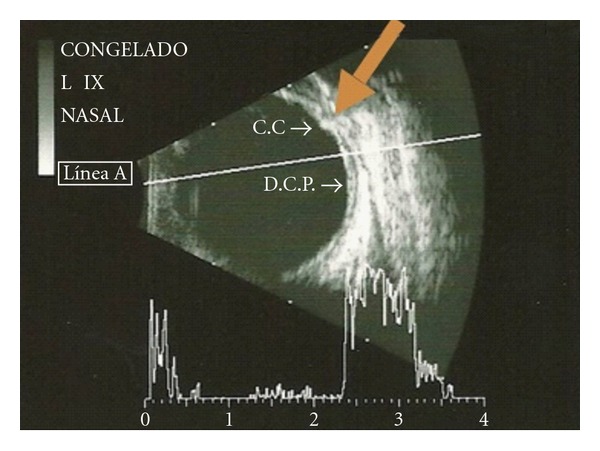
B-scan showing peripheral choroidal effusion (arrow).

A B-scan was performed. 360 degrees choroidal detachment was identified. In the longitudinal cut the choroidal detachment was in the periphery near the ciliary body. Immersion scan showed that the anterior chamber depth was 0.9 mm for the OD, and 1.1 mm for the OS. The axial length of both eyes was 21.8 mm (Figure 1). 

She had been taking topiramate 50 mg a day for migraine 7 days before she started with the symptoms. With the approval of her neurologist, topiramate was discontinued. 

Travaprost 0.005% once a day, Timolol 0.5% BID, and oral acetazolamide 250 mg orally QID were prescribed. Next day uncorrected VA was 20/40 in each eye, her IOP was 10 mm Hg in each eye, and oral acetazolamide was discontinued. One week after the cessation of topiramate her, BCVA was 20/20 with her past hypermetropic refraction, the IOP was 10 mm Hg in each eye, she had no angle synecquias, the anterior chamber was deep, and the optic nerve and the retina were normal.

The anterior chamber depth was 3.05 mm on the right eye and 2.95 mm on the left eye with the A-scan. B-scan showed no ciliochoroidal effusions. Antiglaucoma treatment was discontinued 7 days after the cessation of topiramate. 

## 3. Discussion

We cannot predict an idiosyncratic reaction, but we must be aware of the consequences. Acute angle closure glaucoma is an ophthalmological emergency. Prompt diagnosis and treatment is crucial to maintain healthy eyes after high intraocular pressure has been recognized. 

Some important facts about this entity are idiosyncratic reaction to topiramate can happen at any age, the mechanism is caused by choroidal effusions in contrast to the usual age range, and mechanisms of angle closure glaucoma. The widespread use of topiramate will probably represent an increase in frequency of acute angle closure glaucoma cases, and it is already showing in the literature as case reports [8, 10, 12]. Treatment consists in stopping topiramate and lower the intraocular pressure. Miotics and iridotomies are of no use in this entity. Physicians prescribing topiramate need to be aware of this idiosyncratic reaction for the first 2 weeks or if an increase in the dose is needed. Suspect this entity when there is a myopic shift and acute angle closure glaucoma in a patient on topiramate. Usually around 2 weeks after starting the medication as shown on Table 1. The diagnosis is confirmed by ultrasound with the presence of peripheral choroidal effusion. First step in treatment is to stop topiramate. Topical and systemic antiglaucoma medications are prescribed to lower the IOP. Cycloplegics can help deepening the anterior chamber. Some authors suggest using systemic steroids with the rationale that the ciliary body edema could be caused by inflammation [11].

There is a report of a case with lens-corneal touch that did not improve with topical and systemic treatment, they did a choroidal drainage with resolution of the AACG. In this case the patient was treated initially with pilocarpine that worsened the clinical picture. Miotics are not helpful in this entity [9]. Iridotomies are not useful because there is no pupillary block. Ecography helps us to rule out intraocular tumors or bilateral iris cyst in the periphery that could also produce a bilateral AACG.

## 4. Conclusion

Most reports indicate regression of symptoms and signs when stopping taking topiramate. However, if not recognized as a drug related event, permanent visual loss can occur. It is important to alert physicians and patients using Topiramate for early recognition of signs and symptoms of this entity and prevent permanent damage.

## Figures and Tables

**Table 1:** 

Author	Year	Time taking topiramate
Banta et al. [5]	April 99–July 2001	4 to 21 days (mean 10 days)
Medeiros et al. [8]	2003	5 days and 10 days
Lin et al. [4]	2004	10 days
Parikh et al. [9]	2007	2 weeks
Sbeity et al. [10]	2009	2 weeks
Willett and Edward [11]	2011	10 days
